# Trends in telemedicine use in addiction treatment

**DOI:** 10.1186/s13722-015-0035-4

**Published:** 2015-05-28

**Authors:** Todd Molfenter, Mike Boyle, Don Holloway, Janet Zwick

**Affiliations:** Center for Health Enhancement System Studies, University of Wisconsin–Madison, 4103 Mechanical Engineering Building, 1513 University Avenue, Madison, WI 53706 USA; 16030 Topsail Terrace, Lakewood Ranch, FL 34202 USA; 6201 Chapel Hill Blvd., Plano, TX 75093 USA; 9219 Willard Ct., Urbandale, IA 50322 USA

**Keywords:** Technology implementation, Health information technology, Substance use disorders treatment, Payer strategies

## Abstract

**Introduction:**

Telemedicine use in addiction treatment and recovery services is limited. Yet, because it removes barriers of time and distance, telemedicine offers great potential for enhancing treatment and recovery for people with substance use disorders (SUDs). Telemedicine also offers clinicians ways to increase contact with SUD patients during and after treatment.

**Case description:**

A project conducted from February 2013 to June 2014 investigated the adoption of telemedicine services among purchasers of addiction treatment in five states and one county. The project assessed purchasers’ interest in and perceived facilitators and barriers to implementing one or more of the following telemedicine modalities: telephone-based care, web-based screening, web-based treatment, videoconferencing, smartphone mobile applications (apps), and virtual worlds.

**Discussion and evaluation:**

Purchasers expressed the most interest in implementing videoconferencing and smartphone mobile devices. The anticipated facilitators for implementing a telemedicine app included funding available to pay for the telemedicine service, local examples of success, influential champions at the payer and treatment agencies, and meeting a pressing need. The greatest barriers identified were: costs associated with implementation, lack of reimbursement for telemedicine services, providers’ unfamiliarity with technology, lack of implementation models, and confidentiality regulations. This paper discusses why the project participants selected or rejected different telemedicine modalities and the policy implications that purchasers and regulators of addiction treatment services should consider for expanding their use of telemedicine.

**Conclusions:**

This analysis provides initial observations into how telemedicine is being implemented in addiction services in five states and one county. The project demonstrated that despite the considerable interest in telemedicine, implementation challenges exist. Future studies should broaden the sample analyzed and track technology implementation longitudinally to help the research and practitioner communities develop a greater understanding of technology implementation trends and practices.

## Introduction

Telemedicine applications (apps) that exchange health information from one location to another create new connections between treatment providers and their patients. In general health care, telemedicine is giving patients a sense that their illness is being monitored more closely, the ability to participate in their own health management, and a feeling they have not been forgotten by their doctor [1].

The addiction treatment field offers a promising setting for telemedicine use. The chronic nature of addiction disorders calls for methods for clinicians to stay connected with patients over extended periods of time. Face-to-face contacts between patients and clinicians are limited to scheduled appointments or group sessions. Counselors are not available when they’re most needed: outside the treatment setting, where patients make decisions to stay sober or not. Telemedicine extends the providers’ availability and offers patients an immediate resource.

Telemedicine can increase access to addiction treatment service by removing the barriers of geography and stigma [2]. Patients in rural areas who in the past had to drive long distances can now receive addiction services within their own homes or at a local health care provider. Through telemedicine, patients can also avoid experiencing the perceived stigma of being identified as a patient at a specialty addiction treatment provider. In practice, a variety of electronic modalities are increasing the use of telemedicine in addiction treatment and recovery. The most mature modality, telephone-based care, has been used to provide continuing care for substance use disorders (SUDs) [3]. Continuing care delivered by telephone is easy to implement and does not carry additional costs such as licensing fees or patient training. Telephone-based services have included telephone monitoring, feedback, and counseling. Study results for telephone-based continuing care are mixed, but generally show better results than traditional continuing care [4–6].

An enhanced application of telephone-based technology is interactive voice recognition (IVR) technology. In IVR, an automated telephone system provides patients with different follow-up and feedback options based on their responses to pre-established questions [7]. This technology has been tested in primary care settings with mixed therapeutic results [7, 8]. IVR tested in pilot studies in specialty treatment settings reduced post-outpatient treatment drinking days per week, but only for patients who had achieved treatment goals at time of discharge [9].

Web-based telemedicine services are accessible to many patients and are typically “asynchronous,” meaning that people can access them any time, at their convenience. Several web-based platforms and apps for SUD assessment are available [10, 11]. Overall, web-based telemedicine services have been found to be more effective at reducing alcohol consumed per week than comparison conditions [12, 13]. This is particularly true for nonstudent populations [12]. One population, subcritically injured trauma patients, has not had positive results with web-based telemedicine [14].

For web-based alcohol screening, assessment, and feedback or brief intervention, the Drinker’s Check-up (DCU) has demonstrated positive results in several clinical trials [10, 15]. The DCU (www.drinkerscheckup.com) is an integrated computerized system that includes Moderate Drinking (moderatedrinking.com), a web app for people who want to reduce their drinking, along with several other components: a) a brief screening that utilizes the Alcohol Use Disorders Identification Test (AUDIT); b) an in-depth assessment; c) a full motivational session tailored to the individual’s assessment results; and d) computerized cognitive treatment options that link to web-based mutual aid groups. Similarly, web-based, computerized, brief interventions for drug use demonstrate results similar to or better than clinician-delivered services [16].

An example of a web-based SUD treatment is the Therapeutic Education System (TES) [17]. TES consists of 65 modules based on the Community Reinforcement Approach (CRA) and includes modules on HIV/STD prevention. TES is provided in conjunction with clinician-delivered services and serves as a clinician extender. Research studies are demonstrating superior outcomes for patients using TES compared to treatment as usual. Research studies are also showing TES results comparable to those for the CRA delivered in person by highly trained clinicians [18].

Another web-based treatment approach that has been examined in research studies is Computer-Based Treatment for Cognitive Behavioral Therapy (CBT4CBT) [19]. Research studies have shown positive outcomes (similar to the TES studies) with CBT4CBT compared to treatment as usual or treatment provided solely by clinicians [17, 20].

Videoconferencing for addiction treatment or psychiatry occurs through secure portals on personal computers or dedicated telemedicine consoles. Videoconferencing is currently used in addiction services for: a) video therapy, where patients can interact with clinicians from a remote location or the privacy of their homes; b) recovery supports, where a counselor or peer-support specialist interacts with a person in recovery; and c) specialty services, where patients are placed in contact with hard-to-reach medical specialists, such as an adolescent psychiatrist or a physician who can prescribe buprenorphine. Studies in videoconferencing for addiction services have found no difference in the results or patient satisfaction of care provided in person or by video [21, 22]. Meta-analyses of videoconferencing for psychiatry services have found similar positive results [23, 24], with the notable exception that neither in-person nor video services affected outcomes for post-traumatic stress disorder patients [25].

Mobile devices (smartphones and tablets) make substance abuse treatment and recovery support available 24 h a day, 7 days a week. An early meta-analysis of mobile device use in overall health care determined that it is too early to pool effects of this technology, and that the positive effects that have been realized are primarily attributed to texting interventions within the mobile device apps [26]. Smartphones and tablets offer the same services as telephone-based, web-based, and videoconferencing services. The Addiction Comprehensive Health Enhancement Support System (A-CHESS) app has been found to reduce risky drinking days and to achieve higher abstinence rates than usual care [27]. A-CHESS has also been a useful tool for alcohol relapse prevention for patients following discharge from residential services [28]. A-CHESS is delivered through a smartphone and contains the following key features: a secure discussion board; an “ask an expert” forum; a panic button that provides supportive information; individualized reminders of reasons to not use; automatic messages requesting assistance from people identified as supportive of the patient’s recovery; a GPS-enabled function that sends a warning if a patient is approaching a previously identified high-risk location; a daily check-in assessment of substance use; and a mutual-aid meeting locator. A weekly survey of recovery risk and protection factors is also pushed through the phones, with graphs showing changes over time. Counselors have access to the daily check-in assessment and weekly survey results. Another mobile app called Location-Based Monitoring and Intervention System for Alcohol Use Disorders (LBMI-A) has reduced hazardous drinking days and drinks per day [29]. This app provides numerous features, similar to A-CHESS, for intervening with ongoing drinking, craving, connecting with supportive others, managing life problems, high-risk location alerting, and activity scheduling.

Virtual worlds are an ongoing, synchronous (or real-time) environment facilitated by networked computers that provide a “virtual” social space where people interact and are represented by avatars [29]. Avatars are graphic representations of users that users build with tools supplied in the computerized environment. Users can then control their avatars to interact with others avatars within the virtual environment. Research on virtual reality in addiction treatment that predates virtual worlds and does not allow for synchronous social interaction has established that computer-generated, 3-D environments can simulate reality effectively and provide settings for testing reactions to environmental triggers for craving [30–32].

Virtual worlds and avatars have been used in recent years to address SUDs. Companies design these virtual environments for specific treatment organizations; the resulting virtual world is protected and available only to people with an access code and a password. Services within the virtual worlds may include presentations, individual or group counseling, and a live clinician who interacts with the patients through an avatar. Thus, the virtual world allows synchronous communication between SUD patients and clinicians. To our knowledge, no research studies on the use of virtual worlds to address substance use have been completed; however, early adopters of telemedicine technology in addiction services are considering this option, even though it currently lacks an evidence base.

## Case description

### Case background

In 2011, less than 1% of addiction treatment providers were using telemedicine [33]. Recognizing the increased use of telemedicine in general medicine and the opportune environment for expanding telemedicine services in addiction treatment and recovery, the Substance Abuse and Mental Health Services Administration’s (SAMHSA) Strengthening Treatment Access and Retention State Initiative (STAR-SI) developed a technical assistance program for states interested in using telemedicine for addiction treatment. During 2013–14, the NIATx national program office at the University of Wisconsin-Madison delivered technical assistance focused on providing systems-level and organization change technical assistance to single state authorities (SSAs) and other payers who oversee distribution of state and federal funding for substance abuse treatment programs. The program included five states and one county participant, selected through a competitive review of applicants’ plans for adopting telemedicine. The project served as a real-world laboratory for observing the telemedicine apps that generated the most interest among the participants and for identifying the facilitators and barriers affecting implementation of these apps.

### Case design

Five states (Iowa, Maryland, Massachusetts, Oklahoma, South Carolina) and one county (San Mateo, California) participated. Each participant reviewed a list of telemedicine apps to consider for implementation or expanded use, if implementation had already occurred (Table 1). The list was developed by applying publicly, available evidence-based, patient-interactive technologies used in addiction services and nonevidence-based technologies specifically requested by the states. Use of virtual worlds with avatars was the only nonevidence-based technology that states requested. From this list, each state developed a short list of telemedicine modalities to consider for implementation or expanded use. Then, with the technical assistance, each participant: a) selected the telemedicine modalities to pursue; b) listed the anticipated benefits of the selected telemedicine modalities; and c) developed a plan for implementing or expanding use of the selected telemedicine apps within their state or county, with consideration of anticipated facilitators and barriers to implementing or expanding use.

**Table 1:** Telemedicine modalities & products

Telemedicine modality	Products
Telephone-Based Care	
Post-Treatment Supports	Telephone-Based Continuing Care Program (Using McKay Model) [4]
Web-Based	
Computerized Screening/Brief Intervention	www.drinkerscheckup.com (Drinkers Check-up) [10]
	Screening, Brief Intervention, and Referral to Treatment (SBIRT) [40]
Computerized Treatment	TES [17]
Computerized Treatment Support	Recoveration
Videoconferencing	Dedicated videoconferencing equipment or video interface on personal computer with secure line (e-Psychiatry) [21]
Smartphone Mobile Devices	A-CHESS [27]
Virtual Worlds and Avatars	Virtual worlds developed by Innovation or Second Life

### Measures

For state and county characteristics for participants, the volume of SUD providers and SUD clients served indicates the size of the participant’s provider networks. The baseline telemedicine environment is described by listing the existing telemedicine modalities present, their functions, level(s) of care, and levels of implementation. The study statistics include a listing of telemedicine modalities that the participants selected and the expected benefits of using each modality, as well as the anticipated facilitators and barriers to implementation and expansion.

### Data collection and analysis

State volume statistics were collected through the National Survey of Substance Abuse Treatment Services 2011 dataset [34]. The baseline telemedicine capacity, the telemedicine modalities considered, the telemedicine modalities selected, and the listing of assets and barriers to implementation were collected through monthly reports. During the course of the project (February 1, 2013–June 31, 2014), technical assistance coaches generated monthly reports documenting participant activities. The coaches interacted with the states and county at least once monthly, as they assisted them in telemedicine implementation and expansion. Eisenhardt’s iterative Process of Building Theory from Case Study Research [35] was used to document modality selection as well as facilitators and barriers listed.

### Findings

Variation existed in the number of treatment providers located in each state/county. Maryland had the greatest number of providers, with 362, and San Mateo County had the least, with 25 providers. The number of treatment facilities in South Carolina was 102; there were 145 in Iowa, 218 in Oklahoma, and 328 in Massachusetts. These statistics represent total volume of providers, and are not reflective of the number of users of a certain telemedicine app. Massachusetts had the greatest number of outpatient admissions (45757), and San Mateo had the fewest outpatient admissions (2450). Outpatient admissions from the remaining locations were numbered as follows: South Carolina (13919); Iowa (8663); Oklahoma (16890); and Maryland (39080).

At baseline, telemedicine activity was occurring in four of the six locations (Table 2). Three of Iowa’s facilities were using telephone-based care for treatment and recovery services. Fourteen of Iowa’s 145 facilities were implementing the Recoveration website. This is a pre-programmed website that treatment agencies implement to provide informational supports and counseling services to their consumers. Oklahoma’s e-Psychiatry (or psychiatric videoconferencing) program that services mental health and SUD patients experienced 120000 visits in 2013. Maryland and Massachusetts both had innovative pilots experimenting with virtual worlds and smartphones, respectively.

**Table 2:** Baseline telemedicine activities

State	Technology	Function	Level of care	Level of Implementation (at Baseline)
IA	• Telephone-based care (McKay’s Model)	• Facilitate distance treatment services for problem gambling and substance use disorders.	• Outpatient Treatment (Level I.0 and Level II.1)	Implemented in 14 state-funded programs providing SUD distance treatment.
	• Web-based treatment system	• Provide access to treatment information anytime, anywhere	• Includes:	
			○ Recovery Supports	
	○ www.recoveration.org		○ Relapse Prevention	
	○ Smartphone version of www.Recoveration.org		○ Continuing Care	
			○ Family Education	
MD	Virtual World	Provide access to treatment services	• Outpatient Treatment (Level I.0 and Level II.1)	A pilot (n=7 providers) underway at baseline, with goals to expand
			• Includes:	
			○ Recovery Supports	
			○ Relapse Prevention	
			○ Continuing Care	
MA	Smartphone Mobile Device with A-CHESS	• Provide access to recovery support information anytime, anywhere	• Includes:	4 treatment providers in state were using at beginning of study period.
			○ Recovery Supports	
			○ Relapse Prevention	
			○ Continuing Care	
OK	Video-conferencing for providing psychiatric services	Access to psychiatric assessment, medication management, and consultation	• Outpatient Treatment (Level I.0 and Level II.1)	Conducted 120,000 visits in 2013
San Mateo	None			
South Carolina	None			

Through the project, participants had the opportunity to enhance the provision of telemedicine within their jurisdictions, and several participants selected more than one technology to pursue. The technologies that generated the greatest interest were videoconferencing (n = 4 states) and smartphone mobile devices (n = 3 states) (Table 3). The primary benefits identified for videoconferencing were access to services for rural patients and access to physicians who can prescribe Suboxone® for treatment of opioid dependence. The primary benefit identified for smartphone mobile devices was the ability to reach individuals in treatment and recovery outside the treatment setting. None of the participants chose to adopt or expand use of virtual worlds or telephone-based continuing care.

**Table 3:** Telemedicine modalities and benefits

Payer	Modalities Considered	Modalities Selected	Anticipated Benefits
Iowa	1) Web-based Computerized Treatment System (Recoveration)	Web Portal (Recoveration)	Rural access
			Greater engagement
Maryland	1) Videoconferencing	Videoconferencing (telesuboxone)	Address opiate epidemic
	2) Virtual Worlds		Greater access to physician prescribers
Massachusetts	1) Psychiatric videoconferencing (e-Psychiatry)	Mobile Device (A-CHESS)	Provision of Recovery Support
	2) Virtual Worlds	Web Screening (College Drinker’s Check-up)	Tertiary prevention and harm reduction among college students
	3) Smartphone Mobile Device (A-CHESS)		
	4) Web-based Computerized Treatment System (TES)		
	5) Web Screening (SBIRT and Drinker’s Check-up)		
Oklahoma	1) Smartphone Mobile Devices (A-CHESS)	Smartphone Mobile Device (A-CHESS)	Greater engagement and extension of recovery support
	2) Web Based Computerized Treatment System (TES)	Expand videoconferencing for addiction services	
	3) Web Screen (SBIRT)		
	4) Virtual Worlds		
	5) Psychiatric Videoconferencing (e-Psychiatry)		
San Mateo County	1) Videoconferencing (telepsychiatry, telesuboxone)	Videoconferencing (telesuboxone)	Greater access to physician prescribers
South Carolina	1) Videoconferencing (psychiatry)	Videoconferencing (psychiatry)	Addressing identified disparities in access to specialized SUD care
	2) Smartphone Mobile Devices (A-CHESS)	Smartphone	
	3) Web Based Computerized Treatment (Brief Intervention)	Mobile Devices (A-CHESS)	
	4) Virtual Worlds		Improving collaboration between community partners
			Provision of mobile recovery support

Upon selection of a modality, participants were asked to identify projected facilitators and barriers to adoption of the chosen modality. Perceived facilitators of telemedicine use included having a strong champion, having resources to pay for start-up costs, having established reimbursement mechanisms for telemedicine services, and existing examples of the telemedicine being applied locally or nationally (Table 4). Conversely, participants identified initial and continuing funding for telemedicine services as the greatest barrier to implementation. Other barriers listed included provider and patient resistance, confidentiality concerns, and the absence of implementation models.

**Table 4:** Anticipated facilitators and barriers

State	Technology	Facilitators	Barriers
Iowa	Web-based Computerized Treatment (Recoveration)	Initial funding through SAMSHA TCE Grant	Agency concerns with technology
		NIATx Improvement Collaborative	Agency inexperience with technology
		Treatment agency champion	
Massachusetts	Smartphone Mobile Device (A-CHESS)	Existing example of successful application (A-CHESS)	Identifying start-up funding
	Web Screening (Drinkers Check-up)	Potential case rate funding model	Lack of funding for reimbursement
			Concerns with meeting HIPAA & 42CFR regulations
Maryland	Videoconferencing (telesuboxone)	Strong champion (state governor)	Lack of willing and available MDs for suboxone prescribing
			Limited funding for reimbursement
			Limited models to follow
Oklahoma	Smartphone Mobile Device (A-CHESS)	Smartphone Mobile device start-up funding is available	Limited reimbursement model
	Expanded Videoconferencing	Medicaid expansion covered clinical services for videoconferencing	
San Mateo County	Videoconferencing (telesuboxone)	Demonstrated need for greater MD coverage to address opiate addictions	Competing priorities (ACA implementation)
			Lack of start-up funding
			Lack of funding for reimbursement
			HIPAA compliance concerns
South Carolina	Videoconferencing (telepsychiatry & telesuboxone)	SSA Director Champion	Competing priorities (significant changes in environment)
	Smartphone Mobile Device (A-CHESS)	Psychiatrist/ physician availability	

## Discussion and evaluation

Purchasers of addiction treatment services in this project had an interest in using telemedicine modalities in addiction treatment. The modalities that seemed to create the greatest interest were those that were perceived as readily embraced by treatment providers and their patients.

### Level of research findings and their role in decision**-**making

Meta-analyses support the use of telephone-based continuing care, [5] web-based addiction treatment interventions [12, 13], video-based telemedicine [36], and even smartphone use in mental health [37]. A weakness in these meta-analyses is that they pool studies conducted by the developers. In selecting technologies to consider, the states wanted to know the results of specific products and typically trusted the results reported by developers located in academic settings. However, the states usually wanted to talk to other users of the product and use the product themselves before forming their overall opinions of the technology. The lack of an evidence base for virtual worlds and other considerations affected how the states viewed this modality.

### Other reasons for modality selection

Use of videoconferencing was attractive because it met a specific need: to provide access to a scarce medical resource—buprenorphine (Suboxone), in geographic areas that lack physician prescribers. Videoconferencing was also used in South Carolina to increase access to adolescent psychiatrists in remote or rural areas.

Use of smartphone mobile devices was attractive for a variety of reasons: the apparent low entry costs of equipping patients who already have mobile phones with mobile apps; the ability to create a valuable ongoing relationship with a patient using mobile apps; and the research evidence of their effectiveness [27].

### Implementation considerations

The project identified several issues to consider when implementing telemedicine technology. Among them is the fact that the substance use treatment field lags behind general health care in the use of non-electronic health record (EHR) technologies [33]. As a result, participants in the project experienced a significant learning curve, as they were either just beginning to investigate technology or were in the early stages of implementation.

Implementing technology also changes the traditional workflow, as well as the roles and functions of clinical staff members. Accordingly, treatment organizations will need to develop new workflows and overcome clinical resistance to these changes.

Cost is a significant challenge that states, counties, and providers face in implementing telemedicine. First, start-up costs can be an issue. Despite initial interest, virtual worlds or web-based treatment systems were perceived as too costly to purchase and operate and were not pursued. Second, reimbursement for basic telemedicine services varies broadly between state Medicaid systems and private insurers, with many not reimbursing for these services.

An additional consideration for telemedicine use involves protecting patient anonymity and compliance with the Health Insurance Portability and Accountability Act (HIPAA) and the 42 Code of Federal Regulations (CFR) Part 2 [38]. HIPAA protects the confidentiality and security of health care information. The more restrictive 42 CFR affords special privacy protections to alcohol and drug abuse patient records. Both regulations present an additional challenge when using technology, because no accreditation system documents that a telemedicine system is in compliance. Prospective users must carefully evaluate whether or not the services meet the requirements of these regulations.

### State/county payer and regulatory policy considerations

The participants and the study team considered policies that could promote or hinder the use of the telemedicine modalities piloted in this project.

#### Telephone-based services

There are no licensing, purchasing fees, or equipment costs associated with telephone-based services. The only potential costs are long-distance or cell phone service charges. Clinical staff would need training in delivering brief focused clinical sessions if the telephone-based continuing care model is adopted.

Since telephone-based services involve synchronous (real-time) communication between the clinician and the patient(s), existing individual, group, or case management payment rates could be used for reimbursement. Only policy or rule changes would be required to extend coverage to telephone-based services. For example, Iowa currently reimburses for telephone-based counseling sessions through Substance Abuse Prevention and Treatment block grant funds.

#### Web-based treatment

The lack of payment mechanisms to support the costs of using web-based treatment systems is a major barrier to their adoption by specialty substance use treatment organizations. Because their use involves asynchronous (not in real time) use by the patient, without the immediate involvement of a clinician, the services do not fit the existing fee-for-service reimbursement system. Yet, there are costs to an organization for using computerized treatment, including annual licensing fees, training patients on the use of a system, providing ongoing support as needed, and the clinical time needed to monitor progress reports generated by the system.

If research studies continue to demonstrate effectiveness and future studies show a cost benefit and lower costs per episode of care in using the web-based systems, states may start to experiment with reimbursement models that cover the costs.

#### Videoconferencing

Several policy issues also need to be considered for videoconferencing. First, because platforms are proliferating (and claim to be HIPAA compliant), selecting a platform can be daunting. Second, interstate regulation—when the patient and the counselor are videoconferencing from different states, determining which state regulates the transaction can become complicated. Typically, the state where the patient is located becomes the licensing authority. Hence, the counselor or physician will need to carry a license from the state where the patient is located. Third, at least one state, Florida, offers a certification program for counselors who provide treatment using distance technologies: Certified E-Therapists. Florida’s Certification Board selected the Online Therapy Institutes’ training program. Most states, however, allow licensures achieved for the delivery of in-person clinical care to apply to video care. Lastly, states must allow clinicians providing services through videoconferencing to be reimbursed for those services.

#### Smartphone mobile devices

The lack of payment mechanisms to support services delivered through smartphone mobile devices is a major barrier to their adoption by specialty substance use treatment organizations. Several A-CHESS features use asynchronous technology that does not provide a clinical therapy session. Therefore, the services do not fit the fee-for-service reimbursement system. Costs to an organization for using smartphone mobile devices include annual licensing fees, training patients and staff on the use of a system, providing ongoing support as needed, and the clinical time needed to monitor progress reports generated by the system. Another potential cost, providing smartphones to those who do not have access to them, could result in the cost of providing smartphones and service plans. Fortunately, in some settings carriers and vendors have developed special programs for low- or no-cost services that states, counties, and providers can use to increase access to mobile devices and data plans. As mobile smartphones and computer tablets become more and more ubiquitous, the services could be delivered more affordably to people who have the smartphone mobile devices and adequate service plans.

#### Virtual worlds

Initial costs are an impediment to use of virtual worlds, with implementation cost estimates ranging from $10,000 to $100,000. Few provider organizations can afford costs in the higher range; nor are states likely to support such expensive upfront investments.

Existing virtual worlds such as Second Life could be utilized with lower costs. Second Life will lease use of a virtual “island” that only allows access to those with pass codes. The island has no features, so the environment still has to be created. Second Life provides tools for creating an environment, requiring support from someone with the development knowledge and skills. Also, a person using Second Life can access all but the closed environments, and many existing Second Life environments, such as bars and parties, are not conducive to recovery support.

Since use of the technology involves synchronous (or real-time) communication between the clinician and the patient(s), existing individual and group session payment rates could be used for reimbursement. However, policy or rules would have to be changed to extend coverage to these services.

## Limitations

The analysis has limited generalizability, as it describes the experience of only five states and one county. Yet, the findings offer insights into how purchasers of addiction treatment services are viewing the use of telemedicine for addiction treatment.

Another limitation is that the A-CHESS app was developed by the Center for Health Enhancement Systems Studies at the University of Wisconsin-Madison, where the lead author holds an academic appointment. This could have resulted in natural biases or conflicts of interest regarding this smartphone app. It should be noted, however, that since completion of the state project reported in this study, another evidence-based smartphone app, the LBMI-A [29], has emerged that has many of the same functions as A-CHESS. Hence, the authors propose that the findings related to the states’ interest in the A-CHESS smartphone app may not be product-specific (e.g., A-CHESS) and could be generalized to smartphone apps with similar functionality.

Moreover, not all possible telemedicine apps are addressed. The number of nonevidence-based smartphone mobile apps available for addiction treatment is growing rapidly. Text messaging was included as part of discussions related to smartphone mobile apps and web portals, since these modalities offer that feature. Text messaging should have been discussed as a stand-alone option because it is an accessible, low-cost approach for organizations to provide consumer support, automated content, and reminders [39].

In sum, this analysis would be more complete with: a) inclusion of a greater number of states and territories, b) a larger range of telemedicine modalities, and c) consideration of different implementation models and challenges specific to the different technological modalities and environmental contexts related to institutional settings, reimbursement policy, and levels of care.

## Conclusions

This project did demonstrate considerable interest in telemedicine; facilitators in some states exist; and implementation barriers can interrupt best intentions. These barriers begin with reimbursement challenges at the system level and continue with resistance to using targeted telemedicine at the provider and patient levels. The multilevel nature of telemedicine implementation calls for multilevel models to explain and predict technology adoption.

Finally, new technologies are emerging as potential tools for preventing and addressing addiction. The rapid growth of new technologies requires continual examination of telemedicine technologies to track their use in general addiction treatment practice.

Telemedicine will inevitably play a greater role in addiction treatment and recovery services. Yet, technologies that become part of standard practice will likely be a result of considerations of the technology’s costs, perceived benefits, and ease or difficulty of implementation.

## Abbreviations

A-CHESS: Addiction Comprehensive Health Enhancement Support System
AUDIT: Alcohol Use Disorders Identification Test
DCU: Drinkers Check-up
CET: Certified E-Therapists
CFR: Code of Federal Regulations
CRA: Community Reinforcement Approach
CBT4CBT: Computer-Based Treatment for Cognitive Behavioral Therapy
EHR: Electronic Health Record
HIPAA: Health Insurance Portability and Accountability Act
STAR-SI: Strengthening Treatment Access and Retention State Initiative
SAMHSA: Substance Abuse and Mental Health Services Administration
SSAs: Single State Authorities
SUD: Substance use disorder
TES: Therapeutic Education System

## References

[1] Wang J, Wang Y, Wei C, Yao NA, Yuan A, Shan Y (2014). Smartphone interventions for long-term health management of chronic diseases: an integrative review. Telemed J E Health.

[2] Baca CT, Alverson DC, Knapp-Manuel J, Blackwell GL (2007). Telecounseling in rural areas for alcohol problems. Alcoholism Treat Quart.

[3] Young LB (2012). Telemedicine interventions for substance-use disorder: a literature review. J Telemed Telecare.

[4] McKay JR, Lynch KG, Shepard DS, Pettinati HM (2005). The effectiveness of telephone-based continuing care for alcohol and cocaine dependence: 24-month outcomes. Arch Gen Psychiatry.

[5] McKay JR (2009). Continuing care research: what we have learned and where we are going. J Subst Abuse Treat.

[6] McKay JR, Van Horn DH, Oslin DW, Lynch KG, Ivey M, Ward K (2010). A randomized trial of extended telephone-based continuing care for alcohol dependence: within-treatment substance use outcomes. J Consult Clin Psychol.

[7] Perrine MW, Mundt JC, Searles JS, Lester LS (1995). Validation of daily self-reported alcohol consumption using interactive voice response (IVR) technology. J Stud Alcohol.

[8] Helzer JE, Rose GL, Badger GJ, Searles JS, Thomas CS, Lindberg SA (2008). Using interactive voice response to enhance brief alcohol intervention in primary care settings. J Stud Alcohol Drugs.

[9] Rose GL, Skelly JM, Badger GJ, Ferraro TA, Helzer JE (2015). Efficacy of automated telephone continuing care following outpatient therapy for alcohol dependence. Addict Behav.

[10] Hester RK, Delaney HD, Campbell W, Handmaker N (2009). A web application for moderation training: initial results of a randomized clinical trial. J Subst Abuse Treat.

[11] Moore BA, Fazzino T, Garnet B, Cutter CJ, Barry DT (2011). Computer-based interventions for drug use disorders: a systematic review. J Subst Abuse Treat.

[12] Khadjesari Z, Murray E, Hewitt C, Hartley S, Godfrey C (2011). Can stand-alone computer-based interventions reduce alcohol consumption? A systematic review. Addiction.

[13] Gainsbury S, Blaszczynski A (2011). A systematic review of Internet-based therapy for the treatment of addictions. Clin Psychol Rev.

[14] Neumann T, Neuner B, Weiss-Gerlach E, Tonnesen H, Gentilello LM, Wernecke KD (2006). The effect of computerized tailored brief advice on at-risk drinking in subcritically injured trauma patients. J Trauma.

[15] Squires DD, Hester RK (2004). Using technical innovations in clinical practice: the drinker’s check-up software program. J Clin Psychol.

[16] Ondersma SJ, Svikis DS, Thacker LR, Beatty JR, Lockhart N (2014). Computer-delivered screening and brief intervention (e-SBI) for postpartum drug use: a randomized trial. J Subst Abuse Treat.

[17] Marsch LA, Guarino H, Acosta M, Aponte-Melendez Y, Cleland C, Grabinski M (2014). Web-based behavioral treatment for substance use disorders as a partial replacement of standard methadone maintenance treatment. J Subst Abuse Treat.

[18] Bickel WK, Marsch LA, Buchhalter AR, Badger GJ (2008). Computerized behavior therapy for opioid-dependent outpatients: a randomized controlled trial. Exp Clin Psychopharmacol.

[19] Substance Abuse and Mental Health Services Administration (2013). Results from the 2012 National Survey on Drug Use and Health: Summary of National Findings.

[20] Carroll KM, Ball SA, Martino S, Nich C, Bobuscio TA, Nuro KF (2008). Computer-assisted delivery of cognitive-behavioral therapy for addiction: a randomized trial of CBT4CBT. Am J Psychiatry.

[21] King VL, Stoller KB, Kidorf M, Kindbom K, Hursh S, Brady T (2009). Assessing the effectiveness of an Internet-based videoconferencing platform for delivering intensified substance abuse counseling. J Subst Abuse Treat.

[22] Frueh BC, Henderson S, Myrick H (2005). Telehealth service delivery for persons with alcoholism. J Telemed Telecare.

[23] Hyler SE, Gangure DP, Batchelder ST (2005). Can telepsychiatry replace in-person psychiatric assessments? A review and meta-analysis of comparison studies. CNS Spectr.

[24] Hilty DM, Ferrer DC, Parish MB, Johnston B, Callahan EJ, Yellowlees PM (2013). The effectiveness of telemental health: a 2013 review. Telemed J E Health.

[25] Frueh BC, Monnier J, Yim E, Grubaugh AL, Hamner MB, Knapp RG (2007). A randomized trial of telepsychiatry for post-traumatic stress disorder. J Telemed Telecare.

[26] Free C, Phillips G, Galli L, Watson L, Felix L, Edwards L (2013). The effectiveness of mobile-health technology-based health behaviour change or disease management interventions for health care consumers: a systematic review. PLoS Med.

[27] Gustafson DH, McTavish FM, Chih MY, Atwood AK, Johnson RA, Boyle MG (2014). A smartphone application to support recovery from alcoholism: a randomized clinical trial. JAMA Psychiatry.

[28] Chih MY, Patton T, McTavish FM, Isham AJ, Judkins-Fisher CL, Atwood AK (2014). Predictive modeling of addiction lapses in a mobile health application. J Subst Abuse Treat.

[29] Dulin PL, Gonzalez VM, Campbell K (2014). Results of a pilot test of a self-administered smartphone-based treatment system for alcohol use disorders: usability and early outcomes. Subst Abus.

[30] Saladin ME, Brady KT, Graap K, Rothbaum BO (2006). A preliminary report on the use of virtual reality technology to elicit craving and cue reactivity in cocaine dependent individuals. Addict Behav.

[31] Bordnick PS, Graap KM, Copp H, Brooks J, Ferrer M, Logue B (2004). Utilizing virtual reality to standardize nicotine craving research: a pilot study. Addict Behav.

[32] Culbertson C, Nicolas S, Zaharovits I, London ED, De La Garza R, Brody AL (2010). Methamphetamine craving induced in an online virtual reality environment. Pharmacol Biochem Behav.

[33] Molfenter T, Capoccia VA, Boyle MG, Sherbeck CK (2012). The readiness of addiction treatment agencies for health care reform. Subst Abuse Treat Prev Policy.

[34] Substance Abuse and Mental Health Services Administration: National Survey of Substance Abuse Treatment Services (N-SSATS): 2011 (2012). Data on Substance Abuse Treatment Facilities, BHSIS Series: S-64, HHS Publication No. (SMA) 12–4730.

[35] Eisenhardt KM (1989). Building theories from case study research. Acad Manage Rev.

[36] Backhaus A, Agha Z, Maglione ML, Repp A, Ross B, Zuest D (2012). Videoconferencing psychotherapy: a systematic review. Psychol Serv.

[37] Donker T, Petrie K, Proudfoot J, Clarke J, Birch MR, Christensen H (2013). Smartphones for smarter delivery of mental health programs: a systematic review. J Med Internet Res.

[38] Connors B, Leipold J (2009). The 42 CFR Part 2 and NHIN conundrum. Behav Healthc.

[39] Weitzel JA, Bernhardt JM, Usdan S, Mays D, Glanz K (2007). Using wireless handheld computers and tailored text messaging to reduce negative consequences of drinking alcohol. J Stud Alcohol Drugs.

[40] Vaca FE, Winn D, Anderson CL, Kim D, Arcila M (2011). Six-month follow-up of computerized alcohol screening, brief intervention, and referral to treatment in the emergency department. Subst Abuse.

